# Target parameters and bias in non-causal change-score analyses with measurement errors

**DOI:** 10.1007/s10654-023-00996-4

**Published:** 2023-04-12

**Authors:** Arvid Sjölander, Erin E. Gabriel, Iuliana Ciocănea-Teodorescu

**Affiliations:** 1 Department of Medical Epidemiology and Biostatistics, Karolinska Institutet, Stockholm, Sweden; 2 Section of Biostatistics, Department of Public Health, University of Copenhagen, Copenhagen, Denmark; 3 Victor Babeş National Institute of Pathology, Bucharest, Romania

**Keywords:** Bias, Change-score analysis, Lord’s paradox, Measurement errors

## Abstract

In studies where the outcome is a change-score, it is often debated whether or not the analysis should adjust for the baseline score. When the aim is to make causal inference, it has been argued that the two analyses (adjusted vs. unadjusted) target different causal parameters, which may both be relevant. However, these arguments are not applicable when the aim is to make predictions rather than to estimate causal effects. When the scores are measured with error, there have been attempts to quantify the bias resulting from adjustment for the (mis-)measured baseline score or lack thereof. However, these bias results have been derived under an unrealistically simple model, and assuming that the target parameter is the unadjusted (for the true baseline score) association, thus dismissing the adjusted association as a possibly relevant target parameter. In this paper we address these limitations. We argue that, even if the aim is to make predictions, there are two possibly relevant target parameters; one adjusted for the baseline score and one unadjusted. We consider both the simple case when there are no measurement errors, and the more complex case when the scores are measured with error. For the latter case, we consider a more realistic model than previous authors. Under this model we derive analytic expressions for the biases that arise when adjusting or not adjusting for the (mis-)measured baseline score, with respect to the two possible target parameters. Finally, we use these expressions to discuss when adjustment is warranted in change-score analyses.

## Introduction

In many studies, the aim is to measure the change in a certain score between baseline and follow-up, and to assess how this change-score is associated with a certain covariate measured at or before baseline. A prevailing source of debate is whether or not one should adjust for the baseline score in the analysis; see Glymour et al. [1] and the references therein. In a recent review of change-score analyses in high impact psychology journals, Farmus et al. [2] found that 77% of the included studies had adjusted for the baseline score, whereas the remaining 23% had not. It has been noted that the two analyses may give very different results, and even give different signs of the observed association, which has sometimes been referred to as ‘Lord’s paradox’ [3].

Pearl [4] gave an explanation of this perceived paradox within the modern causal inference framework. He argued that the total causal effect of the covariate on the change-score consists of two parts: a direct causal effect and an indirect causal effect mediated through the baseline score. These may have different signs, in which case the signs of the total and direct causal effects may also differ. He further argued that whether or not adjustment is warranted depends on the choice of target parameter. If the aim is to estimate the direct causal effect, then adjustment is necessary to block the mediating path through the baseline score. However, if the aim is to estimate the total causal effect, then adjustment is harmful since it removes the indirect part of the total effect. Glymour [5] distinguished further between the controlled and natural direct effects, and discussed when and how these can be estimated in change-score analyses.

Although the paper by Pearl [4] is illuminating, his arguments are not applicable to all change-score analyses. In particular, if the aim is to find statistical predictors for the change-score, regardless of whether these have a causal effect on the change-score or not, then the distinction between ‘total’ and ‘direct’ effects is irrelevant for the research question at hand.

When the scores are measured with error, there have been some attempts in the literature to quantify and compare the bias resulting from adjustment for the baseline score or lack thereof. Eriksson and Häggström [6] and Farmus et al. [2] showed that, under a certain statistical model, adjustment for the (mis-)measured baseline score produces an association between a covariate and the (mis-)measured change-score, even if there is no unadjusted (for the true baseline score) association between the covariate and the true change-score. Although correct, this result has two important limitations. First, the statistical model that these authors used is rather restrictive, since it assumes that the true change-score is exactly zero for all individuals, and that there are no systematic errors in the measured scores. Second, these authors tacitly assumed that the target parameter is the unadjusted (for the true baseline score) association between the covariate and the true change-score, thus dismissing the adjusted association as a valid and possibly relevant target parameter.

In this paper we will address these limitations. We argue that, even if the aim is to make predictions rather than to estimate causal effects, there are two possibly relevant target parameters; one adjusted for the baseline score and one unadjusted. We consider both the simple case when there are no measurement errors, and the more complex case when the baseline and follow-up scores are measured with error. For the latter case, we consider a less restrictive model than Eriksson and Häggström [6] and Farmus et al. [2], which allows for both changes in the true scores and systematic measurement errors. Under this model we derive analytic expressions for the biases that arise when adjusting or not adjusting for the (mis-)measured baseline score, with respect to the two possible target parameters. Finally, we use these expressions to discuss when adjustment is warranted in change-score analyses.

To illustrate our points we will use a recently published study by Tajik-Parvinchi et al [7]. We emphasize that, while our theoretical considerations are formulated in terms of our motivating example, our results and conclusions hold more generally in similar change-score studies.

## Motivating example

Tajik-Parvinchi et al. [7] studied 55 children with autism, age 8–12 years. The parents were asked to score their child’s emotion regulation, before and after 10 weeks of treatment with cognitive behavior therapy (CBT). The change in emotion regulation score was regressed on several pre-treatment covariates; here, we will focus on the child’s intellectual ability, which was identified as strongly associated with the change-score. In this analysis, Tajik-Parvinchi et al. [7] did not adjust for the baseline score, stating that such adjustment may ‘result in increased Type I errors’, with reference to Eriksson and Häggström [6] and Farmus et al. [2]

Tajik-Parvinchi et al. [7] clearly stated their aim as non-causal: ‘The present study aimed to identify pre-treatment child characteristics... that *predict* treatment response’ (emphasis added). There are good reasons for this; it would be very difficult to estimate the causal effect, either total or direct, of intellectual ability on response to CBT in practice, since these would most likely be confounded by many unmeasured factors in most realistic settings. Furthermore, it is unclear what practical use one would have of knowing such causal effects, since it is hard to manipulate intellectual ability by intervention. In contrast, it could be of great practical interest to learn whether intellectual ability *predicts* treatment response, since this information could be used to tailor the treatment to those for which it has highest chance of success.

We emphasize that, even though the association between intellectual ability and the change in emotion regulation score may be highly confounded, there may be substantially less confounding of the CBT treatment and the emotion regulation score. By comparing emotion regulation within the same individual, before and after treatment, all time-stable confounders (e.g., sex, genetics) are automatically adjusted for [8, 9]. For pedagogical purposes, we argue as if the change-score in the study by Tajik-Parvinchi et al. [7] were an unbiased measure of the CBT treatment effect, but we note this causal interpretation may be violated by unadjusted time-varying confounders, i.e., predictors of emotion regulation that have different distribution at baseline and follow-up.

## A model for the change-score

Let *P* be the covariate of interest (e.g. intellectual ability), let $U_0$ and $U_1$ be the true baseline score and follow-up score, respectively, and define the change-score

1 $$\begin{aligned} \varDelta U=U_1-U_0. \end{aligned}$$

**Fig. 1:**
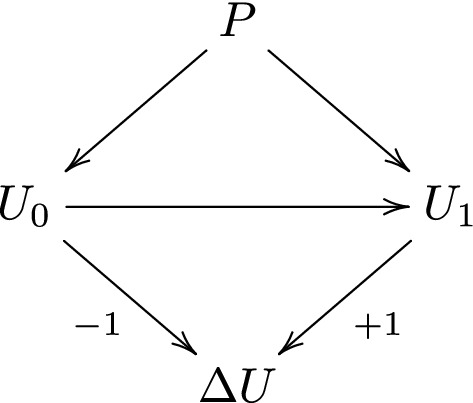
Causal diagram by Pearl [4] for change-score studies

In his discussion of Lord’s paradox, Pearl [4] drew the causal diagram [10, 11] shown in Fig. 1, where *P* is assumed to have a causal effect on both $U_0$ and $U_1$, and $U_0$ is assumed to have a causal effect on $U_1$. The arrows from $U_0$ and $U_1$ to $\varDelta U$, labelled ‘-1’ and ‘+1’, respectively, indicate that $\varDelta U$ is deterministically determined by $U_0$ and $U_1$ through the relation in (1).

**Fig. 2:**
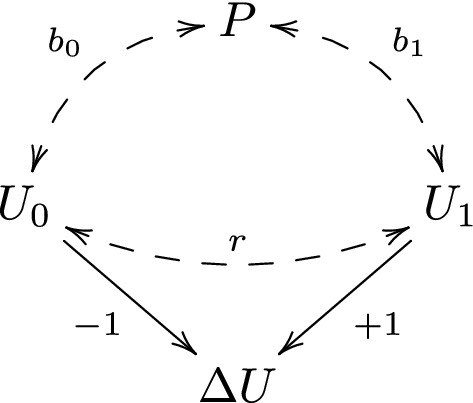
Our assumed path diagram for the study by Tajik-Parvinchi et al. [7]

The causal diagram in Fig. 1 is an unrealistic representation of the study by Tajik-Parvinchi et al. [7], for several reasons. First, one can easily imagine that there is strong unmeasured confounding of both intellectual ability, baseline emotion regulation and follow-up emotion regulation, which hampers causal effect estimation. Second, the direction of causality between intellectual ability and emotion regulation is questionable. Arguably, poor intellectual ability may lead to poor emotion regulation, but also the other way around. To address both these issues we will instead assume the path diagram [12] in Fig. 2, in which the bi-directed dashed arrows between *P*, $U_0$ and $U_1$ represent associations that may be due to a causal influence in either direction, or common causes, or both.

We will assume that $U_0$ and $U_1$ are related to *P* through the linear models

2 $$\begin{aligned} U_0=\, & {} a_0+b_0 P+\epsilon _0\nonumber \\ U_1=\, & {} a_1+b_1 P+\epsilon _1\nonumber \\ \end{aligned}$$

where the error terms $\epsilon _0$ and $\epsilon _1$ are independent of *P*, and normally distributed with mean 0, variance $s^2$ and correlation *r*:

$$\begin{aligned} \left( \begin{array}{c}\epsilon _0\\ \epsilon _1\end{array}\right) \sim N\left\{ \left( \begin{array}{c}0\\ 0\end{array}\right) ,\left( \begin{array}{cc}s^2 &{} s^2r\\ s^2r &{} s^2\end{array}\right) \right\} . \end{aligned}$$

In this model, the difference $a_1-a_0$ is the mean change-score $\varDelta U$ for subjects with intellectual ability $P=0$, and the difference $b_1-b_0$ is the mean increase in change-score $\varDelta U$ corresponding to one unit increase in intellectual ability *P*.

In their bias analyses, Eriksson and Häggström [6] and Farmus et al. [2] considered a special case of model (2) with $a_1=a_0$, $b_1=b_0$ and $r=1$, so that $U_0=U_1$. This is an unrealistically simple model for the study by Tajik-Parvinchi et al. [7], since it implies that the emotion regulation score stays exactly constant across the 10 weeks follow-up for all children.

## Possible target parameters

Consider the aim stated by Tajik-Parvinchi et al. [7]: ‘The present study aimed to identify pre-treatment child characteristics... that *predict* treatment response’. With this aim in mind, a possible regression model of interest could be

3 $$\begin{aligned} \varDelta U = \beta _0+\beta _P P+\varepsilon , \end{aligned}$$

where $\varepsilon$ is an error term. The coefficient $\beta _P$ in this regression model is the mean increase in change-score $\varDelta U$ corresponding to one unit increase in the intellectual ability *P*. This coefficient addresses the question ‘*how much can I expect the effect of CBT treatment to differ between two individuals who differ with one unit in intellectual ability?*’ This may for instance be a relevant target parameter if the study will be used to guide decisions on who will receive the treatment, in future situations where nothing is known about the potential treatment candidates except intellectual ability. A large positive value of $\beta _P$ indicates that individuals with high intellectual ability are likely to benefit more from the treatment than individuals with low intellectual ability. Using standard results for normal distributions (see “Appendix”) we can express $\beta _P$ as

$$\begin{aligned} \beta _P=b_1-b_0, \end{aligned}$$

and we provide an analytic expression for the regression coefficient $\beta _0$ in “Appendix”.

Now, suppose that, in the hypothetical future situation described above, the baseline score $U_0$ would also be available for the treatment candidates. This may be a realistic scenario, since potential treatment candidates may be screened or interviewed before treatment is initiated. It may then be relevant to consider (condition on) this information when deciding who will receive the treatment. To reflect this, we have to modify the regression model as

4 $$\begin{aligned} \varDelta U = \beta _0^*+\beta _P^* P+\beta _{U_0}^*U_0+\varepsilon ^*, \end{aligned}$$

where we have super-indexed the parameters and error term with ‘$*$’, to distinguish them from those in the regression model (3). The coefficient $\beta _P^*$ in this regression model is the mean increase in change-score $\varDelta U$ corresponding to one unit increase in intellectual ability *P*, at a fixed baseline score $U_0$. This coefficient addresses the question ‘*how much can I expect the effect of CBT treatment to differ between two individuals who differ with one unit in intellectual ability, but have the same baseline emotion regulation score?*’ Using standard results for normal distributions (see “Appendix”) we can express $\beta _P^*$ as

$$\begin{aligned} \beta _P^*=b_1-b_0r, \end{aligned}$$

and we provide analytic expressions for the regression coefficients $\beta _0^*$ and $\beta _{U_0}^*$ in “Appendix”.

The distinction between the parameters $\beta _P$ and $\beta _P^*$ under the path diagram in Fig. 2 is analogous to the distinction between the total and direct effect of *P* on $\varDelta U$ under the causal diagram in Fig. 1. The difference between $\beta _P$ and $\beta _P^*$ depends on the correlation *r*. In the extreme (and unrealistic) case where $r=1$, $\beta _P$ and $\beta _P^*$ are equal, which means that the conditioning on baseline score $U_0$ does not alter the amount of information that intellectual ability *P* has about the change-score $\varDelta U$. Apart from this extreme case, $\beta _P$ and $\beta _P^*$ may be very different, which means that the conditioning on baseline score may substantially alter the amount of information that intellectual ability has about the change-score. For instance, suppose that $b_0=b_1=b$. We then have that $\beta _P=0$, which means that we cannot use intellectual ability alone to determine which of two potential candidates who would benefit more from the treatment, without having additional information on the candidates. However if *b* is positive and $r<1$, then $\beta _P^*=b(1-r)$ is positive as well. Thus, if we additionally know that the candidates have equal baseline score, then we may conclude that the candidate with higher intellectual ability is likely to benefit more from the treatment. This is not a ‘paradox’ but reflects the fact that the two parameters $\beta _P$ and $\beta _P^*$ answer different questions, which are both potentially relevant.

We end this section by noting that there are other, equivalent, formulations of models (3) and (4). One such formulation is obtained by ‘moving’ the baseline score $U_0$ to the right-hand side of the equations. Thus, model (3) becomes

5 $$\begin{aligned} U_1 = \beta _0+\beta _P P+U_0+\varepsilon \end{aligned}$$

and model (4) becomes

6 $$\begin{aligned} U_1 = \beta _0^*+\beta _P^* P+(\beta _{U_0}^*+1)U_0+\varepsilon ^*. \end{aligned}$$

We note that the coefficient for $U_0$ in model (5) is fixed to 1. In standard software (e.g., R and Stata), this can be enforced by letting $U_0$ be an ‘offset’ in the model. Another equivalent formulation is obtained by expressing the dependency of the treatment effect on the covariate *P* as an interaction term in the model. Thus, model (3) can be formulated as

7 $$\begin{aligned} U_x = \psi _0+\psi _1x+\psi _2P+\psi _3xP+\varepsilon _x. \end{aligned}$$

Constructing $U_1-U_0$ from the model in (7) gives the model in (3), with $\beta _0=\psi _1$, $\beta _P=\psi _3$ and $\varepsilon =\varepsilon _1-\varepsilon _0$. Similarly, model (4) can be formulated as

8 $$\begin{aligned} U_x = \psi ^*_0+\psi ^*_1x+\psi ^*_2P+\psi ^*_3xP+\psi ^*_4xU_0+\varepsilon ^*_x. \end{aligned}$$

Constructing $U_1-U_0$ from the model in (8) gives the model in (4), with $\beta ^*_0=\psi ^*_1$, $\beta ^*_P=\psi ^*_3$, $\beta _{U_0}^*=\psi ^*_4$ and $\varepsilon ^*=\varepsilon ^*_1-\varepsilon ^*_0$.

## A model for measurement errors in the baseline score and follow-up score

**Fig. 3:**
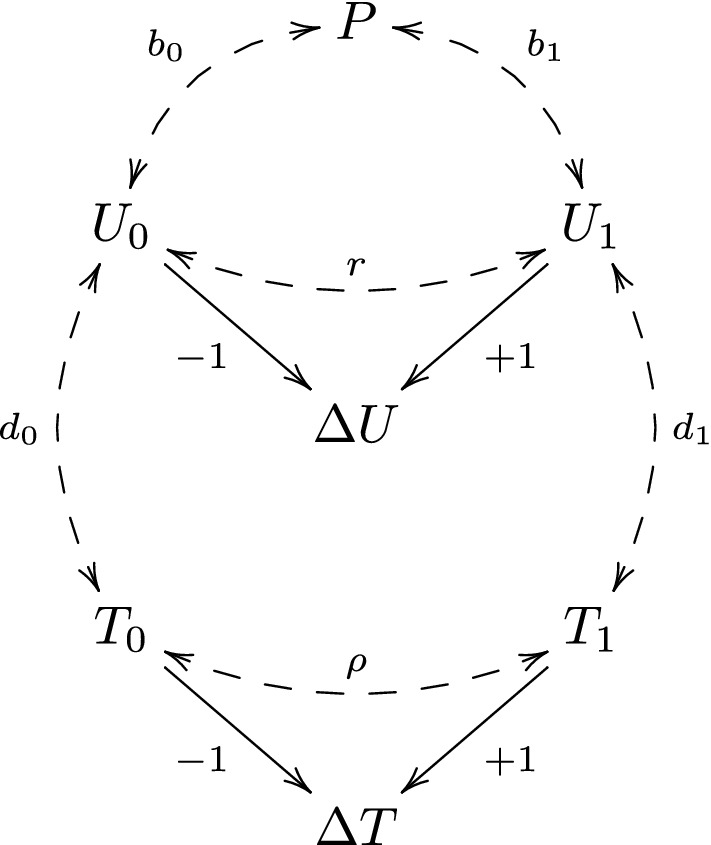
A path diagram illustrating the assumed relations between *P*, $U_0$, $U_1$, $\varDelta U$, $T_0$, $T_1$ and $\varDelta T$

In the study by Tajik-Parvinchi et al. [7], the baseline and follow-up scores were obtained by asking the parents to rate their child’s emotional regulation. Clearly, the rated scores may be subject to measurement errors. To reflect this, we let $U_0$ and $U_1$ denote the true scores, and let $T_0$ and $T_1$ denote the measured scores. As before, $\varDelta U=U_1-U_0$ is the true change-score, and we let $\varDelta T=T_1-T_0$ be the measured change-score. We extend the path diagram in Fig. 2 as in Fig. 3, where we have bi-directed dashed arrows between $U_0$ and $T_0$, between $U_1$ and $T_1$, and between $T_0$ and $T_1$. We expect, of course, that $U_0$ and $U_1$ are associated with $T_0$ and $T_1$. However, this association may be due to both a causal effect and confounding. For instance, parents with high socio-economic status may tend to have children with high emotion regulation, and may also tend to report higher emotion regulation score, irrespective of the true score. If so, then parental socio-economic status would confound the true and measured emotion regulation score.

We assume that $T_0$ and $T_1$ are related to $U_0$ and $U_1$ through the linear models

9 $$\begin{aligned} T_0=\, & {} c_0+d_0U_0+e_0\nonumber \\ T_1=\, & {} c_1+d_1U_1+e_1\nonumber \\ \end{aligned}$$

where the error terms $e_0$ and $e_1$ are independent of $U_0$ and $U_1$, and normally distributed with mean 0, variance $\sigma ^2$ and correlation $\rho$:

$$\begin{aligned} \left( \begin{array}{c}e_0\\ e_1\end{array}\right) \sim N\left\{ \left( \begin{array}{c}0\\ 0\end{array}\right) ,\left( \begin{array}{cc}\sigma ^2 &{} \sigma ^2\rho \\ \sigma ^2\rho &{} \sigma ^2\end{array}\right) \right\} . \end{aligned}$$

In this model, the intercepts $c_0$ and $c_1$ represent a systematic trend across individuals, unrelated to the value of the true score, to overestimate (for positive intercepts) or underestimate (for negative intercepts) the true score. The slopes $d_0$ and $d_1$ represent a systematic trend across individuals to either overestimate large true scores and underestimate true small scores (for slopes $>1$), or the other way around (for slopes $<1$). The error terms $e_0$ and $e_1$ represent errors that are non-systematic across individuals, but possibly systematic within individuals (if $\rho \ne 0$). For instance, if some parents tend to systematically score their children higher than other parents, irrespective of the true score, then we would have a positive correlation in these errors terms ($\rho >0$). In the absence of measurement errors, we have that $T_0=U_0$ and $T_1=U_1$, i.e., that $c_0=c_1=\sigma ^2=0$ and $d_0=d_1=\rho =1$.

Eriksson and Häggström [6] and Farmus et al. [2] considered the special case of model (9) with $c_0=c_1=0$, $d_0=d_1=1$ and $\rho =0$, thus excluding the possibility of systematic measurement errors. In the study by Tajik-Parvinchi et al. [7], emotion regulation was assessed through a standardized assessment tool, which, according to the authors, has previously been evaluated and found to be fairly reliable. Thus, for this study it may be fairly reasonable to assume that there are little or no systematic trends in the measurement errors. However, with less refined assessment tools one can not exclude such systematic trends; hence, we consider both the general model in (9) and the special case of this model obtained by setting $c_0=c_1=0$, $d_0=d_1=1$ and $\rho =0$.

## Estimation and bias

### Possibly systematic measurement errors

In the presence of measurement errors, we cannot directly fit the regression model (3) or (4). We can, however, replace the true scores with the measured scores, thus fitting model

$$\begin{aligned} \varDelta T = \gamma _0+\gamma _P P+{\widetilde{\varepsilon }} \end{aligned}$$

or

$$\begin{aligned} \varDelta T = \gamma _0^*+\gamma _P^* P+\gamma _{T_0}^*T_0+{\widetilde{\varepsilon }}^*. \end{aligned}$$

To assess the bias in the coefficients $\gamma _P$ and $\gamma _P^*$, with respect to the possible target parameters $\beta _P$ and $\beta _P^*$, we use standard results for normal distributions (see “Appendix”), and obtain

$$\begin{aligned} \gamma _P=b_1d_1-b_0d_0 \end{aligned}$$

and

$$\begin{aligned} \gamma _P^*=b_1d_1-b_0d_0\frac{\sigma ^2\rho +d_0d_1s^2r}{\sigma ^2+d_0^2s^2}. \end{aligned}$$

We note that, if $b_0=b_1=b$, $r=1$, $d_0=d_1=1$ and $\rho =0$, as assumed by Eriksson and Häggström [6] and Farmus et al. [2], then the coefficient $\gamma _P^*$ simplifies to $\frac{b\sigma ^2}{\sigma ^2+s^2}$. This is identical to the expression in equation (6) by Eriksson and Häggström [6].

If we consider $\beta _P$ as the target parameter, then, from the expressions above, we have the biases

10 $$\begin{aligned} \gamma _P-\beta _P=b_1(d_1-1)-b_0(d_0-1) \end{aligned}$$

and

11 $$\begin{aligned} \gamma _P^*-\beta _P=b_1(d_1-1)-b_0\frac{\sigma ^2(d_0\rho -1)+d_0^2s^2(d_1r-1)}{\sigma ^2+d_0^2s^2} \end{aligned}$$

for $\gamma _P$ and $\gamma _P^*$, respectively. If we instead consider $\beta _P^*$ as the target parameter, then we have the biases

12 $$\begin{aligned} \gamma _P-\beta _P^*=b_1(d_1-1)-b_0(d_0-r) \end{aligned}$$

and

13 $$\begin{aligned} \gamma _P^*-\beta _P^*=b_1(d_1-1)-b_0\frac{\sigma ^2(d_0\rho -r)+d_0^2s^2r(d_1-1)}{\sigma ^2+d_0^2s^2} \end{aligned}$$

for $\gamma _P$ and $\gamma _P^*$, respectively.

**Fig. 4:**
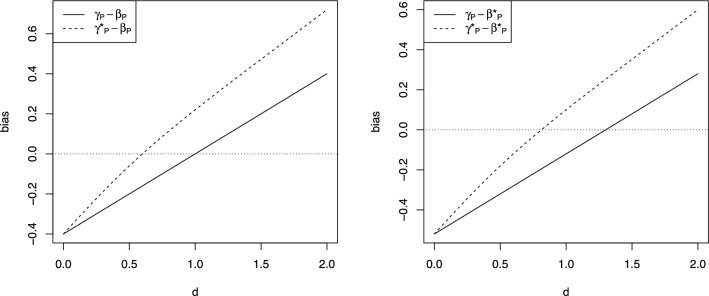
Biases of $\gamma _P$ (solid lines) and $\gamma _P^*$ (dashed lines) with respect to $\beta _P$ (left panel) and $\beta _P^*$ (right panel) as functions of $d_0=d_1=d$, for parameter values $b_0=0.4$, $b_1=0.8$, $s^2=\sigma ^2=1$, $r=0.7$ and $\rho =0.2$

These bias expressions are complex functions of the parameters in models (2) and (9), and there is no general hierarchy between the biases. As an example, Fig. 4 shows the biases of $\gamma _P$ (solid lines) and $\gamma _P^*$ (dashed lines) with respect to $\beta _P$ (left panel) and $\beta _P^*$ (right panel) as functions of $d_0=d_1=d$, for parameter values $b_0=0.4$, $b_1=0.8$, $s^2=\sigma ^2=1$, $r=0.7$ and $\rho =0.2$. We observe that all biases are monotonically increasing in *d*, negative for *d* close to 0 and positive for *d* close to 2. However, the switch from negative to positive bias occurs at different values of *d* for the four combinations of $(\gamma _P,\gamma _P^*)$ and $(\beta _P,\beta _P^*)$. Thus, for some values of *d*, the biases of $\gamma _P$ and $\gamma _P^*$ have opposite signs, so that one of them underestimates the target parameter whereas the other overestimates it. Furthermore, for some values of *d*, the absolute bias of $\gamma _P$ is larger than the absolute bias of $\gamma _P^*$, whereas for other values of *d* it is the other way around.

This example shows that, regardless of whether $\beta _P$ or $\beta _P^*$ is the target parameter, the choice of whether or not one should adjust for the measured baseline score $T_0$ is generally non-trivial, and requires careful thinking about possible values of the model parameters.

### Non-systematic measurement errors

We proceed by considering the important special case when the measurement errors are not systematic, i.e., $d_0=d_1=1$ and $\rho =0$. As argued above, this may be a fairly reasonable model simplification for the study by Tajik-Parvinchi et al [7]. For this special case, the bias expressions in (10)–(13) simplify to

$$\begin{aligned} \gamma _P-\beta _P=0,\\ \gamma _P^*-\beta _P=-b_0\frac{-\sigma ^2+s^2(r-1)}{\sigma ^2+s^2},\\ \gamma _P-\beta _P^*=-b_0(1-r) \end{aligned}$$

and

$$\begin{aligned} \gamma _P^*-\beta _P^*=b_0r\frac{\sigma ^2/s^2}{\sigma ^2/s^2+1}. \end{aligned}$$

Since $\gamma _P$ has zero bias with respect to $\beta _P$, whereas $\gamma _P^*$ generally has non-zero bias, the conclusion is clear: If we consider $\beta _P$ as the target parameter, and we are willing to assume that the measurement errors are not systematic, then we should not adjust for the measured baseline score.

For the target parameter $\beta _P^*$, the conclusion is less trivial. From the expressions above, it follows that $\gamma _P^*$ has smaller absolute bias than $\gamma _P$, with respect to $\beta _P^*$, if

14 $$\begin{aligned} 1-\frac{r}{1-r}\cdot \frac{\sigma ^2/s^2}{\sigma ^2/s^2+1}>0, \end{aligned}$$

but has higher absolute bias otherwise. The left-hand side of this inequality decreases monotonically with the correlation *r* and with the variance ratio $\sigma ^2/s^2$. Thus, if *r* is small, or $\sigma ^2$ is small relative to $s^2$, then the parameter $\gamma _P^*$ is likely to have smaller bias than $\gamma _P$, with respect to $\beta _P^*$.

**Fig. 5:**
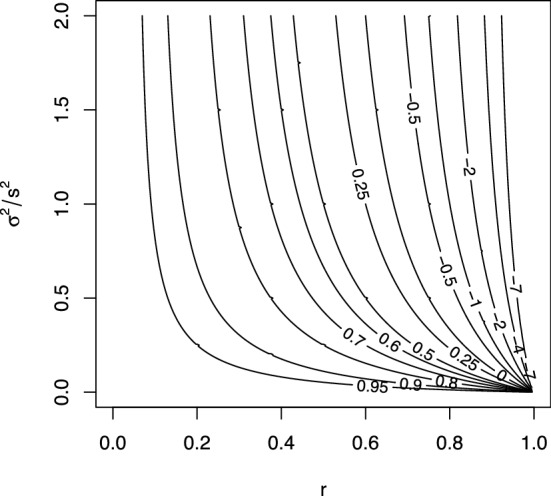
The left-hand side of the inequality in (14) as a function of *r* and $\sigma ^2/s^2$

The contour plot in Fig. 5 shows the left-hand side of the inequality in (14) as a function of *r* and $\sigma ^2/s^2$. We observe that, unless $\sigma ^2/s^2$ is close to 0, the contour lines are close to vertical, so that the left-hand side of the inequality depends mainly on *r*. Specifically, if $\sigma ^2/s^2$ is larger than $\sim 0.5$, then $\gamma _P^*$ has smaller absolute bias than $\gamma _P$ if *r* is smaller than $\sim 0.75$, independently of $\sigma ^2/s^2$. We thus reach the conclusion: if we consider $\beta _P^*$ as the target parameter, and we are willing to assume that (a) the measurement errors are not systematic, (b) $\sigma ^2$ is at least $\sim 50\%$ of $s^2$, and (c) *r* is at most $\sim 0.75$, then we should adjust for the measured baseline score. We emphasize that a violation of the condition in (b) and/or in (c) does not imply that we should not adjust for the measured baseline score, but it implies that the threshold for *r* at which adjustment becomes beneficial depends on the value of $\sigma ^2/s^2$, as seen in the bottom part of Fig. 5.

Whether these assumptions are plausible or not is of course highly context dependent. We don’t have enough subject matter knowledge to firmly judge their plausibility for the study by Tajik-Parvinchi et al. [7]; however, we do suspect that even a standardized assessment tool for emotion regulation may give quite large (non-systematic) measurement errors, and that emotion regulation may vary considerably over 10 week periods within children with autism. If so, then one may tentatively guess that $\sigma ^2/s^2$ was not close to 0 and *r* was not close to 1 in the study by Tajik-Parvinchi et al. [7], in which case the authors would possibly have benefited from adjusting for the measured baseline score, had they been interested in the parameter $\beta _P^*$.

## Discussion

In this paper we have considered a non-causal change-score analysis. We have argued that, just like the causal analysis by Pearl [4], a non-causal analysis may target two different parameters; one adjusted for the baseline score and one unadjusted. We have considered a general model that allows for systematic measurement errors in the baseline and follow-up scores, and under this model we have derived analytic expressions for the biases that arise if one adjusts or doesn’t adjust for the (mis-)measured baseline score, for each of the two target parameters.

We have shown that, if the measurement errors are thought to be non-systematic, then the choice between analyses (adjusting or not adjusting) depends in a relatively straight-forward way on the choice of target parameter and on a small subset of the model parameters. If the measurement errors are thought to be systematic, then the choice between analyses is more difficult, and generally depends on all the model parameters in a complex way. In this case we recommend the analyst to carry out a sensitivity analysis, by varying the model parameters over a grid of plausible values and computing the biases over this grid. At best, this sensitivity analysis reveals that the bias for one of the analysis is consistently smaller than the bias of the other analysis, which then lends support to the least biased analysis. Apart from guiding the choice of analysis, our results can also be used to correct for bias in the estimate obtained from the chosen analysis. This, however, also requires speculation about the model parameters, and would typically be presented as a sensitivity analysis over a grid, rather than as a single bias-corrected estimate.

We have focused on observational studies, in which the baseline score will generally be associated with the covariate of interest. In trials where the covariate is randomized, such associations will disappear, asymptotically. This implies that the coefficient $b_0$ in model (2) is 0, so that the coefficients $\beta _P$ and $\beta _P^*$ in models (3) and (4), respectively, are both equal to $b_1$. Hence, in large randomized controlled trials the adjusted and unadjusted (for the baseline score) analyses will give similar results. However, in small randomzied trials the covariate may be associated with the baseline score by pure chance. In such cases, the adjusted and unadjusted analyses may give different results, and the choice between these may be determined by the same considerations as those outlined in our paper.

The analysis of change-scores is common in several branches of science, including epidemiology, medicine and social science. In such studies, Eriksson and Häggström [6] and Farmus et al. [2] strongly advised against adjustment for the baseline score, unequivocally labeling the adjusted analysis as ‘biased’. We have argued that the degree of bias depends, among other things, on the choice of target parameter, and thus that the advice to not adjust for the baseline score cannot be used as a blanket rule for all studies. We thus hope that our paper may help applied researchers to appreciate the importance of clearly specifying the target parameter, and to think carefully about the appropriate analysis for that parameter.

## Appendix: Supplementary derivations

We have that

$$\begin{aligned} E(U_0|P)=\, & {} E(a_0+b_0 P+\epsilon _0|P)\\= \,& {} a_0+b_0 P+E(\epsilon _0|P)\\= & {} a_0+b_0 P\\ E(T_0|P)=\, & {} E(c_0+d_0U_0+e_0|P)\\= \,& {} E\{c_0+d_0(a_0+b_0 P+\epsilon _0)+e_0|P\}\\=\, & {} c_0+a_0d_0+b_0d_0 P+E(d_0\epsilon _0+e_0|P)\\=\, & {} c_0+a_0d_0+b_0d_0 P\\ Var(U_0|P)= \,& {} Var(a_0+b_0 P+\epsilon _0|P)\\=\, & {} Var(\epsilon _0|P)\\=\, & {} s^2\\ Var(T_0|P)= \,& {} E\{Var(T_0|U_0,P)|P\}\\{} & {} \quad +Var\{E(T_0|U_0,P)|P\}\\=\, & {} E\{Var(c_0+d_0U_0+e_0|U_0,P)|P\}\\{} & {} \quad +Var\{E(c_0+d_0U_0+e_0|U_0,P)|P\}\\= \,& {} E\{Var(e_0|U_0,P)|P\}\\{} & {} \quad +Var\{c_0+d_0U_0+E(e_0|U_0,P)|P\}\\= \,& {} E\{Var(e_0|U_0,P)|P\}+d_0^2Var(U_0|P)\\=\, & {} E(\sigma ^2|P)+Var(a_0+b_0 P+\epsilon _0|P)\\= \,& {} \sigma ^2+d_0^2Var(\epsilon _0|P)\\=\, & {} \sigma ^2+d_0^2s^2\\ Cov(U_0,U_1|P)= \,& {} Cov(a_0+b_0 P+\epsilon _0,a_1+b_1 P+\epsilon _1|P)\\=\, & {} Cov(\epsilon _0,\epsilon _1|P)\\= \,& {} s^2r\\ Cov(T_0,T_1|P)=\, & {} Cov(c_0+d_0U_0+e_0,c_1+d_1U_1+e_1|P)\\=\, & {} d_0d_1Cov(U_0,U_1|P)+Cov(e_0,e_1|P)\\= \,& {} d_0d_1Cov(a_0+b_0 P+\epsilon _0,a_1\\{} & {} \quad +b_1 P+\epsilon _1|P)+\sigma ^2\rho \\= \,& {} d_0d_1Cov(\epsilon _0,\epsilon _1|P)+\sigma ^2\rho \\=\, & {} d_0d_1s^2r+\sigma ^2\rho \end{aligned}$$

By analogous derivations we have that

$$\begin{aligned} E(U_1|P)= & {} a_1+b_1 P\\ E(T_1|P)= & {} c_1+a_1d_1+b_1d_1 P\\ Var(T_1|P)= & {} \sigma ^2+d_1s^2 \end{aligned}$$

Using standard rules for normal distributions we have that

$$\begin{aligned} E(\varDelta U|P,U_0)= & {} E(U_1|P,U_0)-U_0\\= & {} E(U_1|P)+\frac{Cov(U_0,U_1|P)}{Var(U_0|P)}\{U_0-E(U_0|P)\}-U_0 \end{aligned}$$

and

$$\begin{aligned} E(\varDelta T|P,T_0)= & {} E(T_1|P,T_0)-T_0\\= & {} E(T_1|P)+\frac{Cov(T_1,T_0|P)}{Var(T_0|P)}\{T_0-E(T_0|P)\}-T_0. \end{aligned}$$

Using the above relations we finally have that

$$\begin{aligned} E(\varDelta U|P)=(a_1-a_0)+(b_1-b_0)P, \end{aligned}$$

$$\begin{aligned} E(\varDelta U|P,U_0)= & {} a_1-a_0r+(b_1-b_0r)P+(r-1)U_0,\\ \end{aligned}$$

$$\begin{aligned} E(\varDelta T|P)=(c_1+a_1d_1)-(c_0+a_0d_0)+(b_1d_1-b_0d_0)P, \end{aligned}$$

and

$$\begin{aligned} E(\varDelta T|P,T_0)= & {} c_1+a_1d_1-(c_0+a_0d_0)\frac{\sigma ^2\rho +d_0d_1s^2r}{\sigma ^2+d_0^2s^2}\nonumber \\{} & {} \quad +\left( b_1d_1-b_0d_0\frac{\sigma ^2\rho +d_0d_1s^2r}{\sigma ^2+d_0^2s^2}\right) P\\{} & {} \quad +\left( \frac{\sigma ^2\rho +d_0d_1s^2r}{\sigma ^2+d_0^2s^2}-1\right) T_0.\\ \end{aligned}$$
